# Transesophageal Echocardiography and Intracardiac Echocardiography to Guide EVOQUE

**DOI:** 10.1016/j.jaccas.2025.103628

**Published:** 2025-06-11

**Authors:** Nishath Quader, Tsuyoshi Kaneko, Noah Williford, Mark Sintek, Puja Kachroo, Alexander A. Brescia, Harold G. Roberts, Alan Zajarias

**Affiliations:** a Division of Cardiovascular Medicine, Washington University School of Medicine, St Louis, Missouri, USA; b Division of Cardiothoracic Surgery, Washington University School of Medicine, St Louis, Missouri, USA

**Keywords:** 3D, echocardiography, ICE, tricuspid valve

## Abstract

**Objective:**

We demonstrate a step-by-step intraprocedural imaging guide using transesophageal echocardiography (TEE) and intracardiac echocardiography (ICE) to guide EVOQUE in cases with poor intraprocedural imaging.

**Key Steps:**

ICE right ventricular (RV) inflow/outflow view with biplane along with 3-dimensional (3D) multiplanar reconstruction (MPR). TEE gastric views for wire placement. ICE 3D MPR to guide delivery system and device depth. Anchors to leaflet relationship; adequate leaflet capture using 3D ICE. Confirm posterior leaflet capture using 2-dimensional (2D) and 3D TEE gastric images. Atrial and ventricular expansion of device using 3D and 2D ICE. 3D MPR ICE to guide wire out of the RV. Assess final results.

**Potential Pitfalls:**

Frame rate of 3D MPR on ICE is lower than that of TEE, cost of ICE catheters, lack of reimbursement for ICE.

## Graphical Abstract

**Figure:**
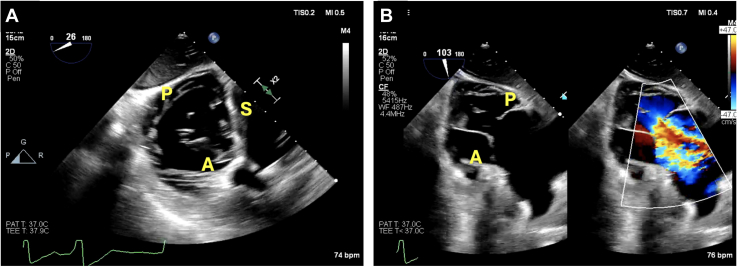

Tricuspid regurgitation (TR) is a cause of significant mortality and morbidity,^1^ and until recently there were no good options for treatment other than diuretics and surgery.^2^, ^3^, ^4^ The TRISCEND data using the EVOQUE device (Edwards Lifesciences) demonstrated significant improvement in TR grade, improvement in functional status and symptoms, leading to FDA approval of the device in 2024.^5^^,^^6^ Unfortunately, one of the exclusions for the TRISCEND trial was poor imaging on transesophageal echocardiography (TEE). Because of this limitation, several patients who may have benefitted from treatment with the device were excluded from the trial. There is growing evidence for the utility of intracardiac echocardiography (ICE) in structural heart disease procedures.^7^ We present 2 cases with severe TR and extremely poor visualization of tricuspid valve (TV) leaflets on TEE who underwent successful EVOQUE placement with the use of simultaneous TEE and ICE for intraprocedural imaging.

**Take-Home Messages**

• Placement of EVOQUE device is feasible with the use of concomitant TEE and ICE in patients with poor standard TEE imaging
• Knowing the limitations of this technique is essential to the success of this procedure and having a safe outcome for patients.

## Case 1

**Figure 1:**
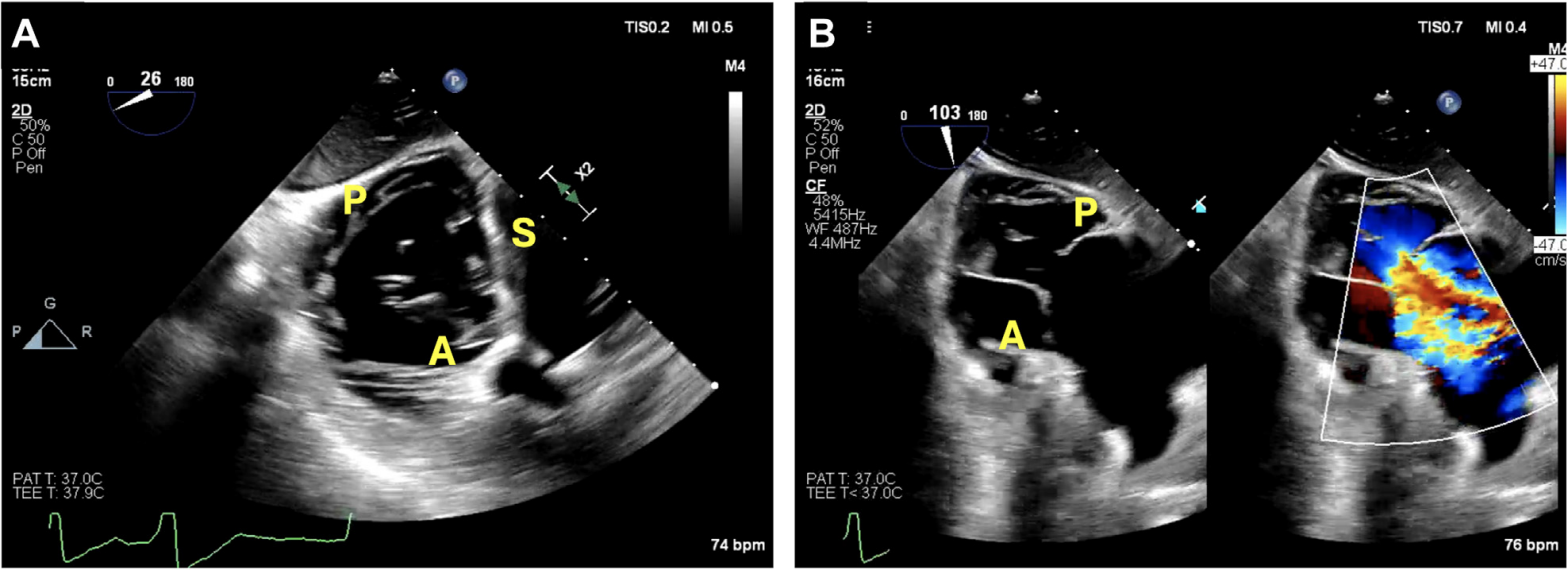
Baseline TEE Imaging (A) Demonstrates short axis view of the tricuspid valve via a gastric image on transesophageal echocardiography. The 3 leaflets, septal (S), anterior (A), and posterior (P), are visualized along with the coaptation gap. (B) The long-axis view of the right ventricle on gastric images shows the posterior and anterior leaflet again with the large coaptation gap along with torrential tricuspid regurgitation.

A 72-year-old man with nonischemic cardiomyopathy, mitral valve repair, permanent pacemaker s/p lead extraction, type I diabetes, cirrhosis, and factor V Leiden mutation presented to our multidisciplinary clinic for evaluation of severe TR. TEE demonstrated severe secondary TR due to annular dilation, and he was deemed ineligible for transcatheter tricuspid edge-to-edge repair (T-TEER) because of the large coaptation gap between his TV leaflets (Figure 1, Video 1). He was deemed anatomically eligible for transcatheter tricuspid valve replacement (TTVR) with the use of EVOQUE, albeit with concerns over the lack of visualization of TV leaflets (Video 1). We used simultaneous TEE and ICE for a successful TTVR, as described below.

### Procedural steps

**Figure 2:**
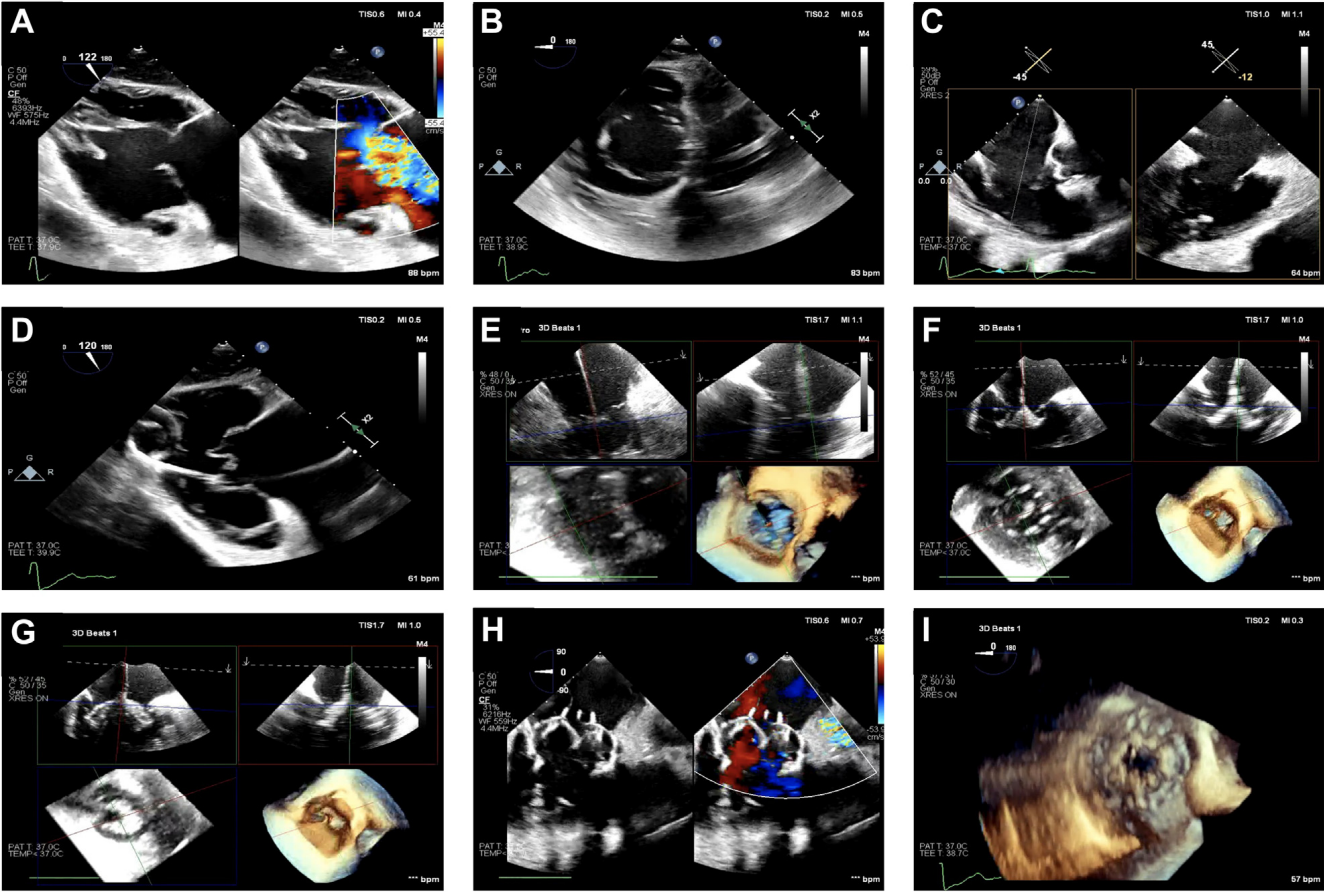
Visual Summary, Providing a Step-by-Step Imaging Guide to the Procedure (A) Baseline gastric long-axis view of the right ventricle (RV) with torrential tricuspid regurgitation (TR). (B) Short-axis view of the tricuspid valve with large coaptation gap. (C) Two-dimensional (2D) intracardiac echocardiography (ICE) demonstrating good visualization of the leaflets. (D) Two-dimensional (2D) transesophageal echocardiography and gastric images to guide wire placement in the RV apex and posteriorly. (E) 3D multiplanar reconstruction (MPR) on ICE being used to maneuver the delivery system into the RV. (F) Device anchors noted on 3D MPR by ICE; also noted are the 9 device anchors in the RV. (G) Ventricular expansion of the device. (H) After atrial expansion, results demonstrate no residual TR. (I) 3D image of the newly implanted EVOQUE device.

**Figure 3:**
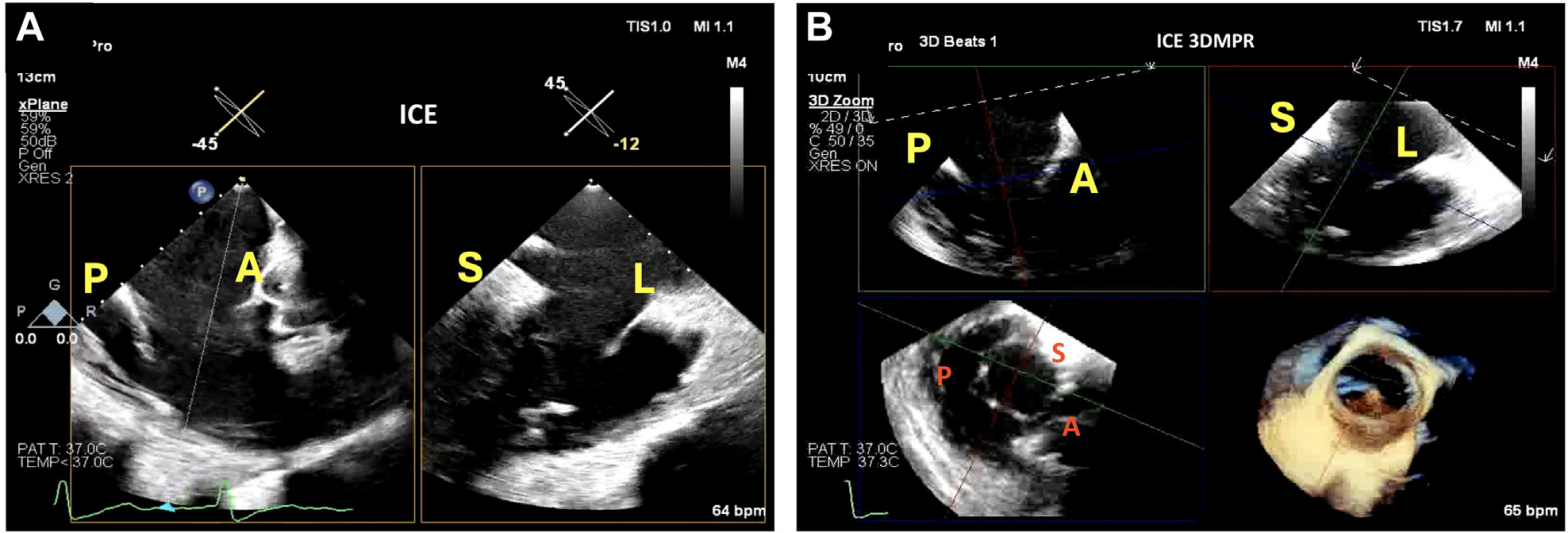
Baseline ICE Imaging (A) 2D ICE showing the biplane of the RV inflow/outflow view. Visualized are the posterior and anterior leaflets along with the septal and lateral commissures. (B) 3D MPR using ICE again demonstrating the tricuspid valve anatomy. In this view, it is critical to visualize the leaflets adequately to ensure visibility during leaflet capture by the anchors. Abbreviations as in Figures 1 and 2.

Figure 2 provides a summary of the imaging steps undertaken intraprocedurally. We used the EPIQ CVx system (Phillips Healthcare) to perform baseline intraprocedural TEE imaging. As part of baseline imaging, we also introduced a 3-dimensional (3D) ICE catheter (VeriSight Pro, Phillips Healthcare) via femoral access into the mid right atrium perpendicular to the annular plane. Although jugular access can be used for ICE, we used femoral access because this may be more suitable for procedural ergonomics for maintaining the stability of the device. We first confirmed that TV leaflet visualization was adequate on the right ventricular (RV) inflow/outflow view with biplane 2-dimensional (2D) and 3D ICE (Figure 3, Video 2).

**Figure 4:**
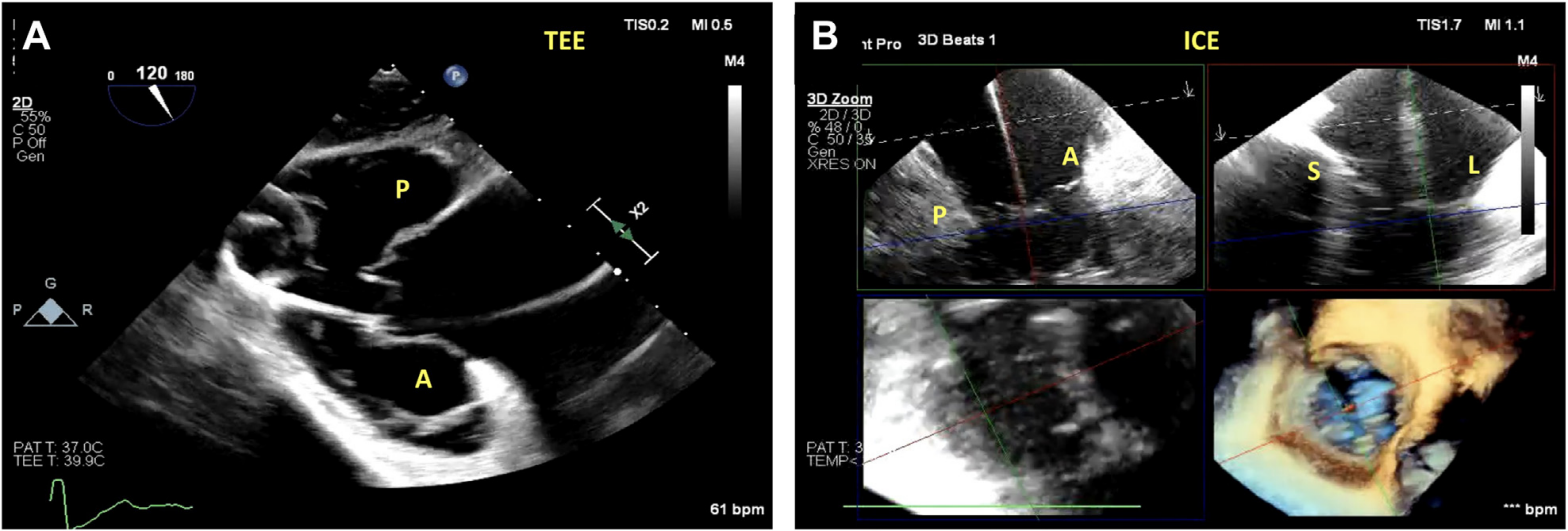
Guiding Wire and Delivery System (A) Gastric image on transesophageal echocardiography (TEE) demonstrating wire placement with the curl of the wire facing posteriorly. (B) 3D MPR on ICE is then visualized to guide the delivery system into the RV considering the anterior, posterior, septal, and lateral orientation of the device. Abbreviations as in Figures 1 and 2.

**Figure 5:**
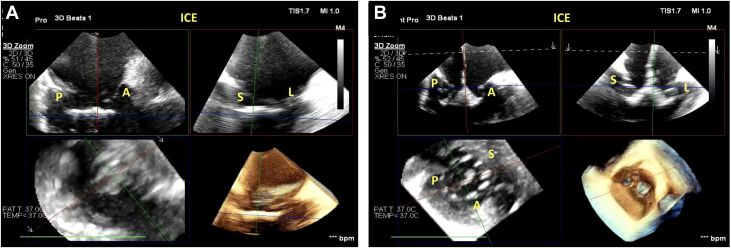
ICE for Leaflet Capture and Visualization of Anchors (A) 3D MPR and ICE is used to visualize the anchors at 90 degrees and their relationship to the tricuspid leaflets. (B) The anchors now pointed atrially, with clear visualization of leaflet capture by the anchor. The 9 anchors are visible in this image, and the green MPR line is used to rotate through each of the anchors to assess leaflet capture. Abbreviations as in Figures 1 and 2.

**Figure 6:**
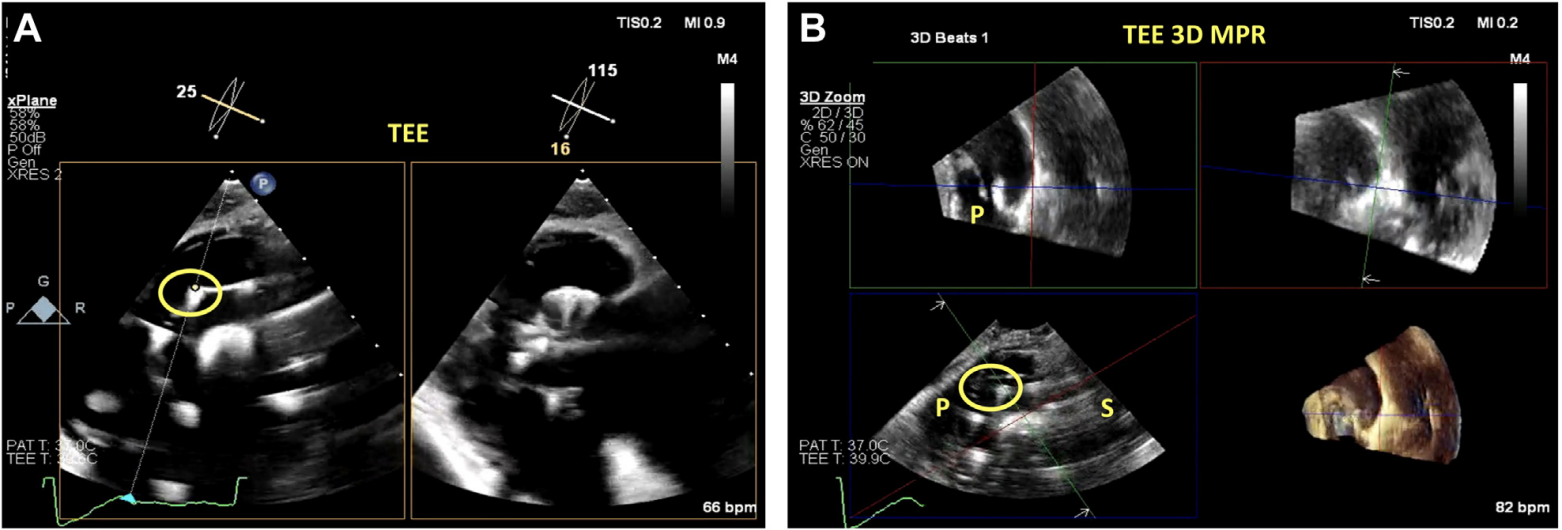
Gastric Images to Visualize Anchors In this case, it was difficult to visualize the posterior anchors owing to acoustic shadowing. Therefore, the gastric views were used on TEE to assess these anchors. (A) Gastric short-axis view of the tricuspid valve on TEE with the anchors, paying special attention to the 10 o’clock anchor (biplane line, yellow dot). (B) 3D MPR using gastric TEE images demonstrates that there is no leaflet capture at the 10 o’clock anchor. This anchor will therefore need to be adjusted to ensure there is leaflet capture. Abbreviations as in Figures 1, 2, and 4.

**Figure 7:**
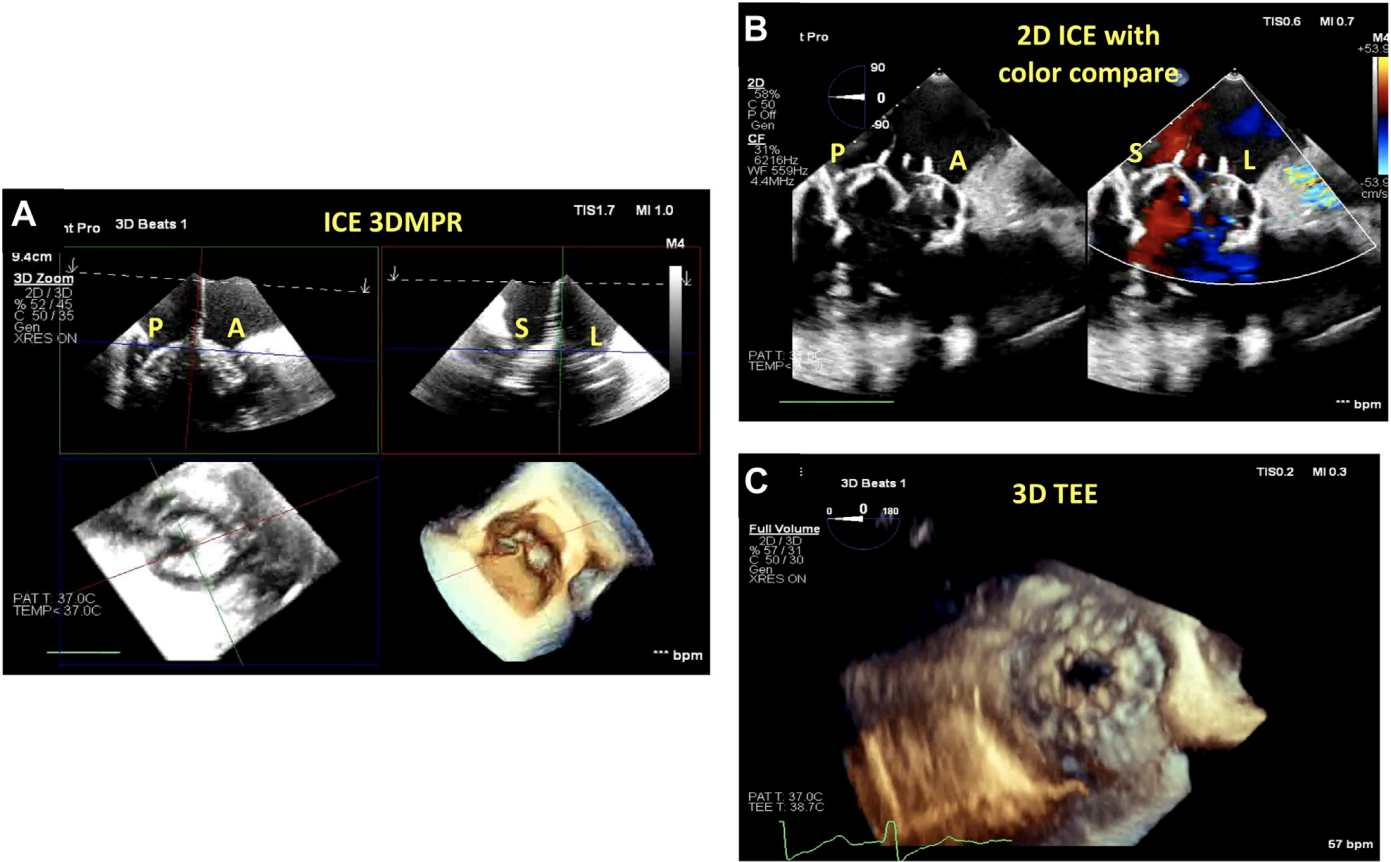
Device Deployment Under ICE (A) After confirmation of leaflet capture by all 9 anchors, the device is expanded ventricularly as can be visualized on the 3D MPR and ICE image. (B) The device is fully expanded with no residual TR. (C) 3D TEE of the newly implanted EVOQUE valve. Abbreviations as in Figures 1, 2, and 4.

Femoral venous access was obtained, and a Safari wire (Boston Scientific) was advanced into the RV apex using the Agilis sheath. 2D TEE gastric views of the long axis of the RV were used for wire placement, ensuring that the curl of the wire was parked in the RV apex, facing posteriorly, and away from the RV papillary muscles (Figure 4A, Video 3A). The EVOQUE delivery system was then brought into the RV; at this time, 2D and 3D ICE was used for delivery system trajectory, visualization of the capsule edge, and assessing of device depth (Figure 4B, Video 3B). Right or left tilt was applied to remove the shadow of the device shaft to visualize the leaflets and the anchors depending on their location. 3D multiplanar reconstruction (MPR) and ICE were then used to visualize the anchors at 90 degrees (Figure 5A, Video 4A) and atrially to assess leaflet capture at the nine anchors of the device (Figure 5B, Video 4B). In this case, visualization of the posterior anchors proved challenging on 3D ICE; therefore, we used the gastric views on TEE (both 2D and 3D) to assess adequate leaflet capture by the posterior anchors (at the 10 o’clock position) (Figure 6, Video 5). Once adequate leaflet capture was again confirmed by means of 3D ICE and gastric imaging, we proceeded with ventricular followed by atrial expansion of the valve (Figure 7A, Video 6); we then used 3D MPR and ICE to guide the wire out of the RV (Video 7A). A final assessment was performed of the valve function, RV function, and pericardial space (Figures 7B and 7C, Videos 7B and 7C).

## Case 2

**Figure 8:**
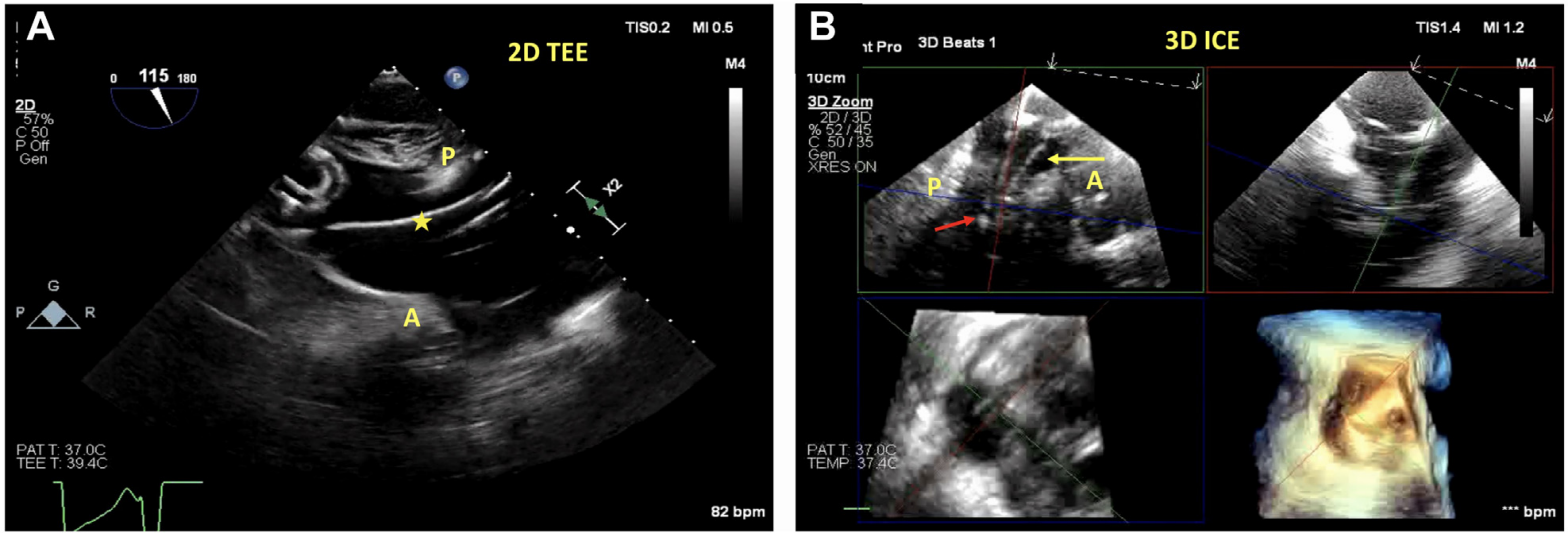
Challenging Intraprocedural Images Because of to Pacemaker Lead (A) The gastric images in this case were challenging because of the pacemaker wire (star). The implanting team should ensure that the coil of the guidewire is again pointed posteriorly. (B) 3D MPR and ICE are challenging, but it is important to differentiate between the pacemaker wire (yellow arrow) and the delivery system. The red arrow shows the device capsule edge in the RV. Abbreviations as in Figures 1 and 2.

**Figure 9:**
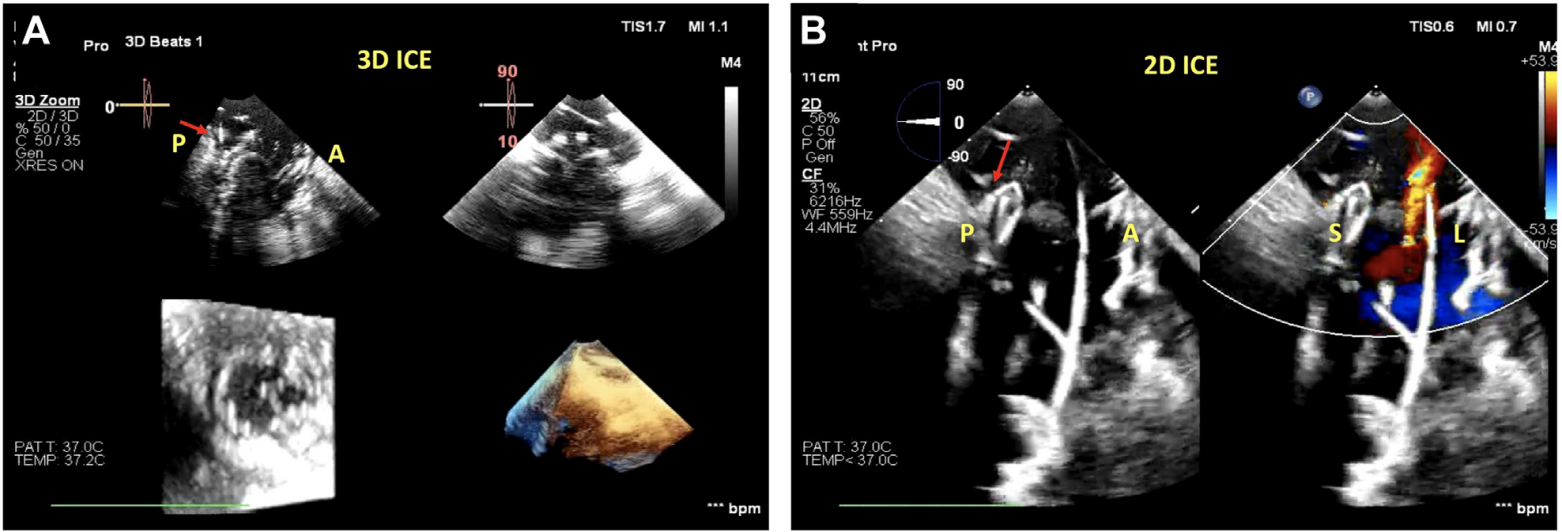
Despite the Difficult Imaging, the Valve Was Successfully Deployed (A) ICE 3D MPR shows the pacemaker wire pinned between the posterior aspect of the valve and the EVOQUE device. (B) 2D ICE also showing the pacemaker wire pinned between the posterior aspect of the native valve and the EVOQUE device. There is now only mild residual valvular TR. Abbreviations as in Figures 1 and 2.

A 67-year-old woman with a history of rheumatic mitral valve disease, mechanical mitral valve replacement, pericardiotomy for constrictive pericarditis, pulmonary hypertension, sick sinus syndrome s/p permanent pacemaker, and atrial fibrillation was referred for evaluation of her heart failure. Her work-up revealed severe TR resulting in multiple heart failure hospitalizations. Her TEE revealed annular dilation along with tethering of her septal leaflet by the pacemaker. She was deemed to be high risk for surgery and ineligible for T-TEER, given the tethered leaflets. She was then evaluated for TTVR: this case was particularly challenging because of the acoustic shadowing from the patient’s pacemaker wire and mitral prosthesis. We used the simultaneous intraprocedural ICE and TEE technique for a successful TTVR. The gastric views were used to ensure that the guidewire was posterior to the pacemaker wire (Figure 8A, Video 8). 3D ICE was used to visualize the capsule edge and to ensure adequate leaflet capture by the anchors (Figure 8B, Video 9). Figure 9 and Video 10 demonstrate the successful deployment of the EVOQUE device with mild central TR and the pacemaker being pinned by the TTVR on the posterior aspect.

## Potential Pitfalls

### Imaging challenges

In patients with poor gastric TEE imaging (or inability to obtain gastric imaging owing to gastric banding or gastric ulcers), the simultaneous ICE and TEE technique may not be feasible, because ICE imaging may not provide adequate imaging of the long axis of the RV and the RV depth. Because of this issue with ICE, the RV apex, along with location of the papillary muscles, would be difficult to visualize, compromising the feasibility of EVOQUE device placement. Owing to excessive amounts of hardware in the right-side chambers, ICE imaging may also be just as challenging as TEE imaging. The team should thoroughly assess visualization of all TV leaflets with the use of ICE before making any attempts at device placement. If any of the leaflets are not well visualized or the gastric views are not available, this technique cannot be attempted. In addition, frame rates of 3D MPR on ICE are lower than that of 3D MPR on TEE; which may add to the difficulty in quickly assessing the delivery system, device anchors, and leaflets.

The implanting team should ensure that their ultrasound equipment is compatible with the ICE catheters and able to provide 3D MPR images of the tricuspid valve; 2D ICE by itself will not be adequate for this procedure. We used the Phillips VeriSight Pro system because we can incorporate this into our TEE imaging platform. In addition, it provides a right and left tilt maneuver that is not available with other systems. There are other ICE systems (Nuvision and Siemens) with unique characteristics, but our experience is limited to the Phillips VeriSight Pro.

### Cost and reimbursement

Procedural costs and reimbursement are a complicating factor in structural heart disease procedures, especially TTVR. Given the Center for Medicare Services reimbursement rate set for TTVR, lack of reimbursement for ICE, cost of the disposable ICE catheters, and extremely low reimbursement for the imager for these complex cases, the additive cost of these procedures needs to be taken into serious consideration. Close collaboration between medical societies, insurance payers, industry, and practicing physicians needs to be established to promote equitable reimbursement reflective of the complexity of the procedural skills. There must be support from the hospital administration to support these extremely complex procedures that are life saving for patients.

## Conclusions

Because experience in TTVR is growing, our center is assessing the best way to image while also reducing procedural times, cost, and improve patient outcomes. The heart team is inclusive of imagers and implanters; a complex procedure such as TTVR requires innovative ways to achieve a successful implant. Even though the implanting physician is placing the ICE catheter in the right atrium, the imaging physician on the implanting team is the one switching back and forth between TEE and ICE along with creating 3D MPR images. The imaging physician should provide the implanter feedback on how to manipulate the ICE catheter to get the appropriate imaging.

3D ICE has been described as an imaging tool during T-TEER,^8^ but we think that the interchangeable use of ICE and TEE provides superior imaging guidance during TTVR in the presence of poor imaging by TEE alone. The decision to use intraprocedural ICE should be established preprocedurally for adequate planning. If there are issues that arise intraprocedurally with poor TEE imaging, the team should have a low threshold to use ICE.

An earlier study has demonstrated early role of 3D ICE in TTVI.^9^ Recently, Hahn et al^10^ published a comprehensive step-by-step guide for TEE imaging during EVOQUE implantation. The present case series is the first description of a step-by-step guide using ICE and TEE during EVOQUE implantation. We demonstrate that TTVR with EVOQUE is feasible even in patients with poor TEE imaging; the implanting team should be facile in concomitant TEE and ICE imaging intraprocedurally. The team also should know the limitations of each of these imaging techniques and when they would not be feasible for TTVR.

## Funding Support and Author Disclosures

Dr Kaneko has served on advisory boards for Edwards Life Science and Johnson & Johnson and has consulted for Medtronic and 4D Medical Anteris. Dr Sintek has consulted for Phillips Healthcare, Edwards Life Sciences, Boston Scientific, Biotronik, and Shockwave. Dr Zajarias has consulted for Edwards Life Sciences, Medtronic, and Anteris. All other authors have no reported that they have no relationships relevant to the contents of this paper to disclose.

## Appendix

Video 1A (A) Midesophageal transesophageal echocardiography (TEE) imaging demonstrating poor visualization of the tricuspid valve leaflets. (B) Gastric TEE images demonstrating large coaptation gap with torrential tricuspid regurgitation.

Video 1B (A) Midesophageal transesophageal echocardiography (TEE) imaging demonstrating poor visualization of the tricuspid valve leaflets. (B) Gastric TEE images demonstrating large coaptation gap with torrential tricuspid regurgitation.

Video 2A (A) Gastric transesophageal echocardiography, showing a short-axis view of the tricuspid valve with the septal (S), posterior (P), and anterior (A) leaflets. (B) 3D multiplanar reconstruction using intracardiac echocardiography (ICE), showing the the P, A, S, and lateral (L) annulus. (C) 2D ICE of the RV inflow/outflow view.

Video 2B (A) Gastric transesophageal echocardiography, showing a short-axis view of the tricuspid valve with the septal (S), posterior (P), and anterior (A) leaflets. (B) 3D multiplanar reconstruction using intracardiac echocardiography (ICE), showing the the P, A, S, and lateral (L) annulus. (C) 2D ICE of the RV inflow/outflow view.

Video 2C (A) Gastric transesophageal echocardiography, showing a short-axis view of the tricuspid valve with the septal (S), posterior (P), and anterior (A) leaflets. (B) 3D multiplanar reconstruction using intracardiac echocardiography (ICE), showing the the P, A, S, and lateral (L) annulus. (C) 2D ICE of the RV inflow/outflow view.

Video 3A (A) Placement of the guidewire with the curl oriented posteriorly and in the RV apex. (B) 3D multiplanar reconstruction and intracardiac echocardiography used to guide the delivery system into the RV. This view also helps in assessing trajectory.

Video 3B (A) Placement of the guidewire with the curl oriented posteriorly and in the RV apex. (B) 3D multiplanar reconstruction and intracardiac echocardiography used to guide the delivery system into the RV. This view also helps in assessing trajectory.

Video 4A (A) 3D multiplanar reconstruction (MPR) and intracardiac echocardiography, demonstrating the anchors at 90 degrees; note the relationship of the anchors to the leaflets. (B) Further expansion of the anchors that are now visualized on the short-axis view. This view is essential is assessing leaflet capture by each of the anchors. The MPR planes are rotated through each anchor to assess leaflet capture.

Video 4B (A) 3D multiplanar reconstruction (MPR) and intracardiac echocardiography, demonstrating the anchors at 90 degrees; note the relationship of the anchors to the leaflets. (B) Further expansion of the anchors that are now visualized on the short-axis view. This view is essential is assessing leaflet capture by each of the anchors. The MPR planes are rotated through each anchor to assess leaflet capture.

Video 5A (A) Because of poor visibility of the posterior anchors, gastric view on transesophageal echocardiography (TEE) was used to assess these anchors. Biplane imaging through the 10 o’clock anchor shows inadequate leaflet capture. (B) This is further confirmed by 3D multiplanar reconstruction of the gastric TEE image showing that the posterior leaflet has not been captured by the anchor.

Video 5B (A) Because of poor visibility of the posterior anchors, gastric view on transesophageal echocardiography (TEE) was used to assess these anchors. Biplane imaging through the 10 o’clock anchor shows inadequate leaflet capture. (B) This is further confirmed by 3D multiplanar reconstruction of the gastric TEE image showing that the posterior leaflet has not been captured by the anchor.

Video 6A (A) Ventricular expansion of EVOQUE. (B) Atrial expansion using 3D multiplanar reconstruction and intracardiac echocardiography.

Video 6B (A) Ventricular expansion of EVOQUE. (B) Atrial expansion using 3D multiplanar reconstruction and intracardiac echocardiography.

Video 7A (A) 3D multiplanar reconstruction and intracardiac echocardiography (ICE) used to guide the wire out of the RV after device deployment. Final assessment of the EVOQUE device using. (B) 2D ICE and (C) 3D transesophageal echocardiography.

Video 7B (A) 3D multiplanar reconstruction and intracardiac echocardiography (ICE) used to guide the wire out of the RV after device deployment. Final assessment of the EVOQUE device using. (B) 2D ICE and (C) 3D transesophageal echocardiography.

Video 7C (A) 3D multiplanar reconstruction and intracardiac echocardiography (ICE) used to guide the wire out of the RV after device deployment. Final assessment of the EVOQUE device using. (B) 2D ICE and (C) 3D transesophageal echocardiography.

Video 8A (A) Gastric transesophageal echocardiography images demonstrate the pacemaker wire. The guidewire should be placed in a way to allow the coil to point posteriorly. (B) Final placement of the guidewire.

Video 8B (A) Gastric transesophageal echocardiography images demonstrate the pacemaker wire. The guidewire should be placed in a way to allow the coil to point posteriorly. (B) Final placement of the guidewire.

Video 9 Excessive acoustic shadowing noted on 3D multiplanar reconstruction and intracardiac echocardiography. It is important to differentiate between the pacemaker wire and the delivery system. The red arrow points to the device capsule edge in the right ventricle.

Video 10A (A) 3D multiplanar reconstruction and intracardiac echocardiography (ICE), showing that the valve is deployed with pinning of the pacemaker wire between the EVOQUE device and the posterior tricuspid annulus. (B) 2D ICE also demonstrating the pacemaker wire pinned against the posterior annulus. The patient has mild residual tricuspid regurgitation.

Video 10B (A) 3D multiplanar reconstruction and intracardiac echocardiography (ICE), showing that the valve is deployed with pinning of the pacemaker wire between the EVOQUE device and the posterior tricuspid annulus. (B) 2D ICE also demonstrating the pacemaker wire pinned against the posterior annulus. The patient has mild residual tricuspid regurgitation.

## References

[1] Topilsky Y., Maltais S., Medina Inojosa J. (2019). Burden of tricuspid regurgitation in patients diagnosed in the community setting. JACC Cardiovasc Imaging.

[2] Dhoble A., Zhao Y., Vejpongsa P. (2019). National 10-year trends and outcomes of isolated and concomitant tricuspid valve surgery. J Cardiovasc Surg (Torino).

[3] Dreyfus J., Audureau E., Bohbot Y. (2022). TRI-SCORE: a new risk score for in-hospital mortality prediction after isolated tricuspid valve surgery. Eur Heart J.

[4] Kundi H., Popma J.J., Cohen D.J. (2019). Prevalence and outcomes of isolated tricuspid valve surgery among medicare beneficiaries. Am J Cardiol.

[5] Kodali S., Hahn R.T., George I. (2022). Transfemoral tricuspid valve replacement in patients with tricuspid regurgitation: TRISCEND study 30-day results. JACC Cardiovasc Interv.

[6] Grayburn P.A., Kodali S.K., Hahn R.T. (2024). TRISCEND II: novel randomized trial design for transcatheter tricuspid valve replacement. Am J Cardiol.

[7] Tang G.H.L., Zaid S., Hahn R.T. (2025). Structural heart imaging using 3-dimensional intracardiac echocardiography: JACC: Cardiovascular Imaging position statement. JACC Cardiovasc Imaging.

[8] Hamid N., Aman E., Bae R. (2024). 3D Navigation and intraprocedural intracardiac echocardiography imaging for tricuspid transcatheter edge-to-edge repair. JACC Cardiovasc Imaging.

[9] Wang L., Petrossian G., Robinson N. (2024). Early experience of 3-dimensional intracardiac echocardiography in transcatheter tricuspid interventions. J Soc Cardiovasc Angiogr Interv.

[10] Hahn R.T., Makkar R., Makar M. (2024). EVOQUE tricuspid valve replacement system: state-of-the-art screening and intraprocedural guidance. JACC Cardiovasc Interv.

